# 2330. Ventilation Strategies Based on an Aerodynamic Analysis During a Large-Scale SARS-CoV-2 Outbreak in an Acute-Care Hospital

**DOI:** 10.1093/ofid/ofad500.1952

**Published:** 2023-11-27

**Authors:** Se Yoon Park, Jungyeon Yu, Jin Su Song, Shin Young Lee, Jin Hwa Kim, Yeon Su Jeong, Sun Mi Oh, Tae Hyong Kim, Sanghwan Bae, Eunjung Lee

**Affiliations:** Hanyang University College of Medicine, Yongin, Kyonggi-do, Republic of Korea; Korea Institute of Civil Engineering and Building Technology, Goyang-si, Kyonggi-do, Republic of Korea; Handong Global University, Pohang-si, Kyongsang-bukto, Republic of Korea; Korea Diseases Control and Prevention Agency, Sejong-si, Kyongsang-namdo, Republic of Korea; Soonchunhyang University Seoul Hospital, Seoul, Seoul-t'ukpyolsi, Republic of Korea; Soonchunhyang University Seoul Hospital, Seoul, Seoul-t'ukpyolsi, Republic of Korea; Soonchunhyang University Seoul Hospital, Seoul, Seoul-t'ukpyolsi, Republic of Korea; Division of Infectious Diseases, Department of Internal Medicine, Soonchunhyang University Seoul Hospital, Seoul, Seoul-t'ukpyolsi, Republic of Korea; Korea Institute of Civil Engineering and Building Technology, Goyang-si, Kyonggi-do, Republic of Korea; Soonchunhyang University Seoul Hospital, Seoul, Korea, Seoul, Seoul-t'ukpyolsi, Republic of Korea

## Abstract

**Background:**

The severity of coronavirus disease-2019 (COVID-19) has decreased owing to antiviral agents and herd immunity; however, transmission among hospitalised patients could be fatal. Therefore, strategies to prevent the spread of COVID-19 in medical institutions are crucial. This study aimed to investigate ventilation strategies to prevent nosocomial transmission of COVID-19.

**Methods:**

We retrospectively conducted an epidemiological investigation of the severe acute respiratory syndrome coronavirus 2 (SARS-CoV-2) outbreak between 12 February and 5 March 2021 in a referral teaching hospital in Korea. The pressure difference and air change per hour (ACH) of the rooms were measured in the largest outbreak ward. Airflow dynamics were measured based on the opening or closing of the outside window and door by using an oil droplet generator, indoor air quality sensor, and particle image velocimetry in the index patient’s room, corridor, and opposite rooms.

**Results:**

During the outbreak, 283 COVID-19 cases were identified. The largest outbreak occurred at the 8^th^ floor followed by the 9^th^ and 7^th^ floors of the main building. According to an epidemiological investigation, the SARS-CoV-2 spread occurred sequentially from the index room to the nearest room, especially the opposite. The aerodynamic study demonstrated that droplet-like particles in the index room diffused through the corridor and the opposite room through the opening door (Figure 1). The mean ACH of the rooms on the 8^th^ floor was 1.44; the air supply volume was 15.9% larger than the exhaust volume, forming a positive pressure. Compared with the open-door condition, droplet-like particles did not diffuse into an adjacent room facing each other in the closed-door condition. The concentration of droplet-like particles in the ward was lowered during natural ventilation, and their spread to an adjacent room facing the corridor was also reduced (Figure 2).

Location of the index patient and detection sensor of the droplet-like particles

**Figure:**
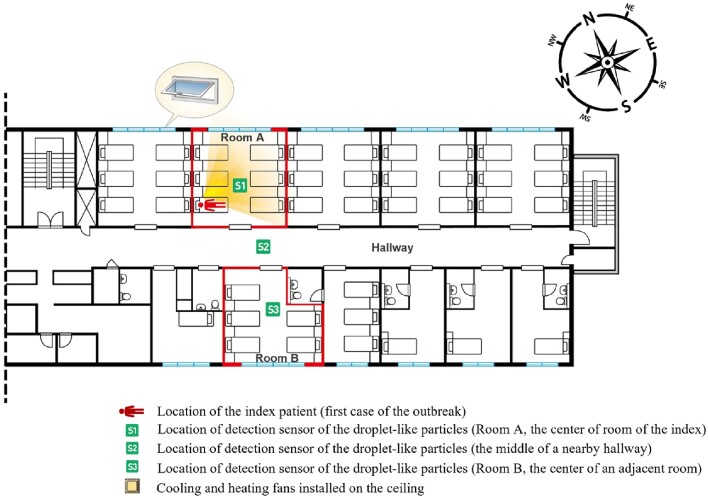

Relative ratio in measurement of droplet-like particles, PM2.5, concentration according to natural ventilation conditions

**Figure:**
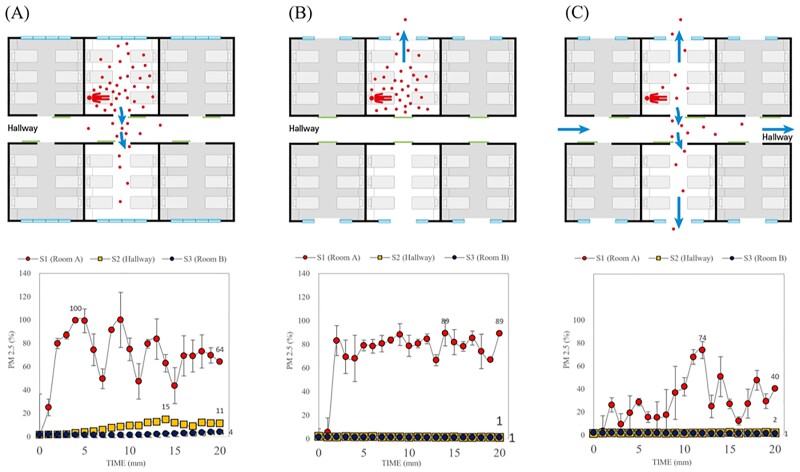

(A) Window closed/indoor-open (usual condition: ventilation with the heating, ventilation, and air conditioning [HVAC] system without natural ventilation), (B) Window open/indoors closed (condition for improvement: close the door and perform natural ventilation for each room), (C) Window and indoor opening (condition for improvement: opening all doors and windows in all the rooms and maximising natural ventilation).

**Conclusion:**

The spread of droplet-like particles between rooms could be attributed to the pressure difference between the rooms and corridor. To prevent the spread of transmission between rooms, increasing the ACH in the room by maximising natural and mechanical ventilation is essential.

**Disclosures:**

**All Authors**: No reported disclosures

